# Amino acid metabolic reprogramming: future prospects for cholangiocarcinoma therapy

**DOI:** 10.1038/s41420-025-02843-9

**Published:** 2026-01-09

**Authors:** Sijia Hua, Fan Fei, Jiawen Li, Yuting Liu, Yuhong Gao, Xiang Wang, Xiulin Dong, Qiang Liu, Jianfeng Yang

**Affiliations:** 1 Zhejiang Chinese Medical University, Hangzhou First People’s Hospital. No. 548 Binwen Road, Binjiang District, Hangzhou, 310053 Zhejiang Province China; 2 Hangzhou Normal University, No.120 Jinhua Road, Gongshu District, Hangzhou, 310015 Zhejiang Province China; 3 Department of Gastroenterology, Affiliated Hangzhou First People’s Hospital, School of Medicine, Westlake University, No. 261 Huansha Road, Hangzhou, 310006 Zhejiang China; 4 Key Laboratory of Integrated Traditional Chinese and Western Medicine for Biliary and Pancreatic Diseases of Zhejiang Province, Hangzhou, 310006 China; 5 Hangzhou Institute of Digestive Diseases, Hangzhou, Hangzhou, 310006 China; 6 Zhejiang University School of Medicine, Hangzhou, 310058 China

**Keywords:** Cancer metabolism, Cancer therapy

## Abstract

Cholangiocarcinoma (CCA) is a highly heterogeneous disease with a poor prognosis and a 5-year survival rate of less than 20% due to late diagnosis and limited therapeutic options, and the current problems in the treatment of CCA can be mainly attributed to the low rate of early diagnosis, the limited availability of targeted drugs, and the gradual increase in chemoresistance. Metabolic reprogramming in CCA causes the accumulation of large amounts of lactic acid and glycolytic intermediates, exacerbating hypoxia and the formation of an acidic environment at the tumor site, which further reduces the effectiveness of therapeutic drugs. Amino acid metabolic reprogramming promotes the proliferation, metastasis, spreading, and tumor angiogenesis of CCA cells, and some amino acid metabolites, in turn, regulate the metabolic state and gene expression of cells, which in turn regulates the cellular phenotype. Abnormal metabolism of amino acids negatively affects the progression of CCA. In the amino acid metabolism of CCA, the PI3K/AKT/mTOR and AMPK/Nrf2 pathways are two key pathways, and c-Myc plays an important role in glutamine metabolism as a transcription factor. Future studies should design targeted drugs around the abnormal accumulation process of glutamine, arginine and other amino acids to disrupt the amino acid uptake dominance in malignant tumors, as well as design novel drugs according to the changes in the tumor microenvironment.

## Facts

- 5-hydroxytryptamine levels are dysregulated in cholangiocarcinoma (CCA) patients, with increased bile serotonin levels and altered expression of enzymes involved in its synthesis and degradation. Serotonin promotes CCA cell growth and angiogenesis.
- Tumor cells rely on glutamine for energy metabolism through the TCA cycle, supported by the AMPK/Nrf2 signaling axis. Elevated expression of lysyl oxidase-like 1 promotes angiogenesis through the FAK/MAPK axis.
- Serine and threonine metabolism are active in CCA tissues, supporting tumorigenesis. Defects in enzymes like argininosuccinate synthase are associated with reduced tumor cell proliferation.
- Inhibitors of amino acid transporters, such as nanvuranlat, have demonstrated improved progression-free survival in patients with advanced CCA. Enzymes like methionine adenylyltransferase 1A and histone lysine acetyltransferase KAT2B are potential therapeutic targets.
- Mutations in IDH1/2 drive metabolic changes that inhibit immune responses. Targeting these metabolic pathways, either through small-molecule inhibitors or combination therapies, may restore immune function and enhance the efficacy of immunotherapy.

## Open questions

- How does amino acid metabolic reprogramming in cholangiocarcinoma (CCA) interact with immune cells in the tumor microenvironment, thereby affecting the efficacy of immunotherapy? Are there potential immune-metabolic targets that can enhance the effects of immunotherapy by modulating amino acid metabolism?
- How do the PI3K/AKT/mTOR and AMPK/Nrf2 pathways work together to regulate amino acid metabolism in cholangiocarcinoma (CCA)? Are there specific metabolic nodes that can be targeted to simultaneously inhibit these two pathways, thereby more effectively suppressing tumor growth and the development of chemoresistance?

## Introduction

Currently, the incidence of CCA, especially iCCA, is gradually increasing worldwide [1–4]. Owing to its difficult early diagnosis and limited therapeutic options, CCA often has a low 5-year survival rate and a high recurrence rate [5]. Moreover, when CCA is detected, it is often in advanced stages, at which point patients have lost the opportunity for early surgery. Although systemic therapy for CCA has evolved in recent years, patient survival is still limited to less than 1 year after treatment [6]. Chemotherapy, as a first-line treatment for patients with unresectable CCA, is limited in clinical application because of the unpredictable resistance of tumor cells, and the combination of chemotherapeutic agents has emerged as an available avenue to ameliorate resistance, but has the risk of further enhancing cancer cell resistance [7, 8]. The role of immunotherapy in CCA has also been further emphasized on the basis of studies of the immune microenvironment of CCA tumors. For patients who have failed first-line treatment, there are currently targeted therapies for specific sites such as IDH, FGFR, EGFR, C-Met, and BRAF. The mutation rates of the metabolic enzymes isocitrate dehydrogenase 1 (IDH1) and isocitrate dehydrogenase 2 (IDH2) in ICC patients are ~22–28%, while the mutation rate in ECC is around 10%. Similarly, the reported mutation rate of the epidermal growth factor receptor (EGFR) in ICC ranges from 0 to 20%. Therefore, targeted therapy targeting these two mutations has become a promising new systemic treatment option for CCA [9–12]. Furthermore, combination therapies such as combined immunotherapy, immunotherapy combined with chemotherapy, and immune checkpoint inhibitors combined with anti-angiogenic agents have shown disappointing efficacy in advanced CCA, especially in patients with metastatic CCA [13–16]. Overall, current treatments for CCA have produced disappointing results, highlighting the need for novel therapeutic strategies.

Although the tumor microenvironment varies among cancers, metabolic reprogramming allows all tumor cells to use extracellular nutrients to produce intracellular macromolecules (such as proteins, lipids, and nucleotides) that facilitate nutrient transfer to the intracellular compartment [17]. Cancer cells have been shown to utilize different sources of nutrients, including intermediates produced by the tricarboxylic acid (TCA) cycle as precursors of lipids, amino acids or nucleotides that support tumor progression [18]. In general, cancer cells have considerable flexibility in the use of energy sources, depending on the environmental availability of nutrients and the heterogeneity of cell populations within the tumor [19].

To compete effectively for energy and survive in the harsh nutritional conditions in the tumor microenvironment, cancer cells need to be more tolerant and metabolically efficient than normal cells. Therefore, the metabolic and reprogramming status of cancer cells varies at each step of cancer progression [20]. The metabolic pathways that reprogram cancer cells are related mainly to bioenergetics and anabolism [21]. Adenosine triphosphate (ATP) is produced in cells through two main pathways: glycolysis and mitochondrial oxidative phosphorylation. Most malignant cells present the “Warburg” effect, which causes large amounts of lactic acid and glycolytic intermediates to accumulate in the tumor cells, thereby creating an acidic environment that drives tumor progression [22]. This phenomenon largely contributes to tumor acidosis, which in turn synergistically promotes tumor progression and resistance to certain antitumour therapies and compromises antitumour immunity, and is currently an attractive therapeutic target [23, 24]. Other sources of energy can be synthesized within the tumor cell or taken up from the environment and converted into biosynthetic intermediates for anabolism [25]. Other tumor cells rely on the autophagic process to obtain amino acids for biosynthesis, and autophagy is usually accompanied by an increase in reactive oxygen species (ROS) production in the cell [26].

At present, tumor metabolic targets can mainly be divided into three aspects: targeting tumor cells, targeting the tumor microenvironment, and regulating systemic metabolism [27]. When used in combination with the existing treatment methods, the amino acid consumption strategy can play a greater role. When used in combination with chemotherapy, the consumption of amino acids can induce normal cells to enter the cell cycle arrest stage, protecting them from the damage of chemotherapy and increasing the attack on cancer cells [28]. Furthermore, for drug-resistant cells, amino acid depletion therapy helps counteract the intracellular mechanisms of chemoresistance and reduces patients’ dependence on chemotherapy drugs. Compared with other therapies, amino acid depletion therapy is safer and causes less damage to normal cells [29].

## Amino acid metabolic reprogramming and signal regulatory network

**Fig. 1:**
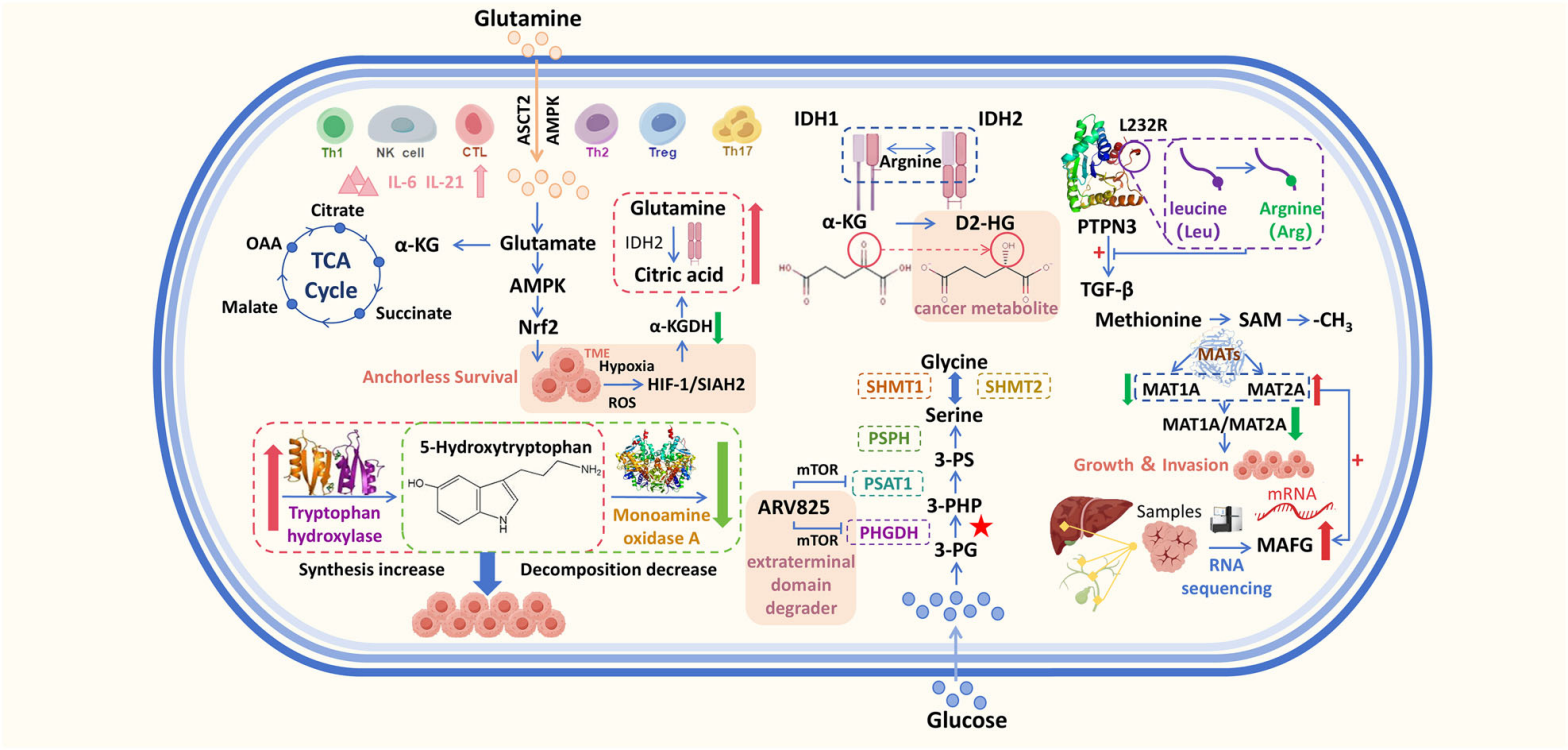
Metabolism and rearrangement of amino acids in CCA. Glutamine fuels the TCA cycle via conversion to glutamate (by GLS/GLS2) and α-ketoglutarate, supporting anchorage-independent cancer cell survival through the AMPK/Nrf2 axis. Hypoxic and acidic tumor microenvironments activate HIF-1, shifting glutamine metabolism from oxidative to reductive carboxylation. Cytokines like IL-6 and IL-21 also modulate glutamine transporter expression. IDH1/2 mutations convert α-ketoglutarate to D2-HG, while PTPN3 L232R mutations are common in iCCA. 5-HT, synthesized from tryptophan, promotes tumor proliferation, invasion, and angiogenesis, making its synthesis a key therapeutic target in CCA. CCA tissues show elevated oxidative stress and increased serine/glycine levels. PHGDH upregulation in iCCA directs glucose toward serine synthesis, with PHGDH and PSAT1 being key enzymes in the SGOC pathway; their inhibition induces epigenetic metabolic suppression. MAT generates SAM, a methyl donor for intracellular reactions. Differential expression of MAT1A/MAT2A correlates with tumor progression, while elevated MAFG and MAT2A predict poor prognosis in CCA and HCC. TCA tricarboxylic acid, OAA oxalacetic acid, ASCT2 alanine-serine-cysteine transporter 2, TME tumor microenvironments, D2-HG D-2-hydroxyglutarate, α-KG α-ketoglutarate, PTPN3 protein tyrosine phosphatase nonreceptor 3, SAM *S*-adenosylmethionine, MAFG MAF bZIP transcription factor G, PHGDH phosphoglycerate dehydrogenase, PSAT1 phosphoserine aminotransferase 1, SHMT1 human serine hydroxymethyltransferase 1, SHMT2 human serine hydroxymethyltransferase 2, 3-PG 3-phosphoglycerate, 3-PHP 3-phosphohydroxypyruvic acid, 3-PS 3-phosphatidylserine (p-serine), MAT methionine adenosyltransferase, SIAH2 Siah E3 ubiquitin protein ligase 2 gene.

The reprogramming of amino acid metabolism in CCA affects the proliferation, metastasis, spreading and tumor angiogenesis of CCA cells at different levels, while the metabolites produced by amino acid metabolism also affect the surrounding microenvironment of the cancer cells, which in turn affects the metabolic state of the cells and gene expression. The relationship between different types of amino acids and their changes in cholangiocarcinoma and the phenotype of cholangiocarcinoma is shown in Table 1, while the effect of abnormal amino acid metabolism on the phenotype of cholangiocarcinoma is explained through a summary of the mechanism diagram (Fig. 1).

**Table 1:** Relationship between the expression of different kinds of amino acids in CCA and the phenotype of CCA.

Amino acids	Key protein	Experimental content	Experimental conclusions	Ref.
Glutamine	Glutaminase (GLS,GLS2)	Glutamine is converted to glutamate by GLS/GLS2 and then to α-ketoglutarate to replenish the TCA cycle substrate.	Cancer cells rely on glutamine metabolism to maintain ATP levels under anchorage-independent conditions.	[30]
	AMPK/Nrf2	AMPK activation induces Nrf2 and its target proteins, promoting glutamine catabolism to maintain energy homeostasis.	AMPK supports cancer cell survival via the AMPK/Nrf2 signaling axis.	[30]
	ASCT2	AMPK induces the expression of ASCT2, increasing glutamine uptake.	Increased ASCT2 expression enhances glutamine consumption but may lead to glutamine depletion in cancer cells.	[35]
	IL-6, IL-21	Cytokines in the tumor immune microenvironment (IL-6 and IL-21) upregulate the expression of glutamine transporter proteins.	These cytokines enhance glutamine metabolism by promoting the expression of amino acid transporter proteins.	[131, 132]
	HIF-1, α-KGDH, SIAH2	Hypoxia and acidosis activate HIF-1, reducing α-KGDH activity and promoting SIAH2 ubiquitination.	This leads to a shift in glutamine metabolism from oxidative carboxylation to reductive carboxylation.	[133]
Arginine	Argininosuccinate Synthase	Argininosuccinate synthase acts as a tumor suppressor by converting citrulline to arginine.	Defects in this enzyme correlate with decreased arginine levels and tumor cell proliferation.	[60, 61]
	Arginine Deiminase	ADI degrades arginine, and its pegylated form (ADI-PEG20) has shown promise in phase I-II clinical trials.	ADI-PEG20 is a potential anticancer therapy but requires further validation in phase III trials.	[60, 62]
	IDH1, IDH2	Mutations in IDH1 and IDH2 convert α-ketoglutarate to D-2-hydroxyglutarate (D2HG), a cancer metabolite.	These mutations are associated with tumor progression in CCA.	[134]
	PTPN3 (L232R mutation)	The L232R mutation in PTPN3 enhances TGF-β signaling and blocks its tumor suppressor function.	This mutation is common in iCCA and promotes tumor progression.	[135]
	LOXL1	LOXL1 binds to αvβ3 integrin, promoting angiogenesis in iCCA via the FAK/MAPK axis.	LOXL1 depletion inhibits tumor cell proliferation, metastasis, and angiogenesis.	[136]
Tryptophan	Tryptophan Hydroxylase, Monoamine Oxidase A	Increased serotonin levels in bile due to elevated tryptophan hydroxylase and decreased monoamine oxidase A.	Serotonin promotes CCA cell growth and is a potential therapeutic target.	[63, 66]
Serine	PHGDH	PHGDH is upregulated in iCCA, promoting glucose metabolism towards serine synthesis.	PHGDH is a potential therapeutic target for iCCA.	[55]
	BET PROTAC degrader (ARV825)	Synergistic anticancer effect of ARV825 with genetic ablation of mTOR pathway members.	Inhibition of PHGDH and PSAT1 leads to epigenetic inhibition of metabolism.	[56]
Methionine	MAT1A, MAT2A, MAFG	Dysregulation of the MAT gene expression affects SAM levels and tumor progression.	High SAM expression is associated with low MAFG expression, while MAT2A overexpression increases MAFG promoter activity.	[45–47, 53]

This table presents the key proteins involved in the metabolism of specific amino acids and their impact on the phenotype of CCA. It includes experimental content, experimental conclusions, and references.

### Glutamine metabolism-signaling pathways in CCA

Glutamine, one of the more important amino acids in energy metabolism, is involved in the growth and proliferation of tumor cells. Tumor cells have undergone metabolic reprogramming and exhibit an increased dependence on glutamine for the TCA cycle [30]. Zhang et al. [31] conducted plasma metabolic analysis and found that the glutamine content in the metabolites of ICC patients was significantly elevated. Another study, after performing spatial metabolite analysis on CCA tissue samples, suggested that the tumor core region exhibited severe glutamine depletion, and CCA cells demonstrated a strong “addiction” to glutamine [30]. A clinical study that included 431 patients demonstrated that glutamine synthetase (GS) expression status can be used as a predictor of HCC prognosis and the efficacy of hepatic resection combined with targeted therapy [32]. On this basis, Zhang et al. [31] revealed that glutamine reversed iron death induced by ALK5 overexpression and that the intestinal flora influenced cancer development by altering the above signaling axis in ICC. This result mirrors the conclusion drawn by Chen [33] that a decrease in the extracellular glutamine concentration increases the sensitivity of cells to apoptosis. Moreover, cholangiocarcinoma cells that are resistant to chemotherapeutic drugs, such as cisplatin-resistant cholangiocarcinoma cells, exhibit accelerated cell cycle progression. This leads to an increased demand for nutrients by cancer cells and consequently induces reprogramming of amino acid metabolism, with alterations in glutamine metabolism being particularly critical. In addition to the effective reduction of cancer progression markers in resistant cells by glutamine deprivation alone, studies have found that the combination of Glutaminase 1(GLS1) inhibition and glucose transporter (GLUT) inhibition, that is, simultaneous inhibition of glutamine and glucose uptake, more effectively reverses cisplatin resistance in cholangiocarcinoma cells [34]. This suggests that targeting glutamine metabolism may hold promise for overcoming chemotherapeutic drug resistance in cholangiocarcinoma cells, thereby enhancing the efficacy of chemotherapy.

In further exploring the mechanisms underlying the changes in glutamine concentration in cholangiocarcinoma, we have found that these changes are associated with multiple signaling pathways. After entering the cell, glutamine is converted to glutamate by glutaminase (GLS/GLS2) and converted to α-ketoglutarate by dehydrogenation and transamination, thus backfilling the substrate of the TCA cycle. Cancer cells primarily utilize glutamine-derived tricarboxylic acid cycle energy metabolism to maintain ATP levels in the absence of anchoring conditions, and this process requires interaction with cystine to inhibit excessive oxidative stress. The anchorage-independent survival of cancer cells described above is supported primarily by glutamine catabolism via the adenosine 5’-monophosphate (AMP)-activated protein kinase (AMPK)/nuclear factor erythroid 2-related factor 2 (Nrf2) signaling axis. Specifically, AMPK activation induces Nrf2 and its target proteins, allowing cancer cells to maintain energy metabolism homeostasis solely through periglutaminolysis. In contrast, AMPK is concurrently involved in inducing the expression of the glutamine transporter protein alanine-serine-cysteine transporter 2 (ASCT2), increasing glutamine consumption, which can induce glutamine deficiency in cancer cells [35]. In addition, the tumor immune microenvironment is usually a hypoxic and acidic environment, which leads to the activation of HIF-1 and the accumulation of lactate. The activation of HIF-1 promotes ubiquitination within cancer cells, while overexpression of the biomarker annexin A1 (ANXA1) can recruit the deubiquitinating enzyme USP5 to stabilize key enzymes in glutamine metabolism, synergistically enhancing glutamine uptake and glutamate production, and alleviating oxidative stress at the tumor site to inhibit tumor growth [36]. c-Myc is a proto-oncogene and transcription factor in organisms that is overexpressed in a variety of malignant tumors and has been reported to be associated with tumor initiation, progression and maintenance in CCA [37–40]. Glutamate further generates ATP through the TCA cycle. One study revealed a close link between c-Myc, through the regulation of miRNAs, and glutamine metabolism as well as energy and reactive oxygen species homeostasis [41]. Overexpression of miR-181c in CCA leads to reduced levels of NDRG2 [42]. Another study elucidated that NDRG2, as a tumor suppressor gene, is negatively regulated by lipid carrier protein 2 (LCN2) in CCA cells [43]. NDRG2 promotes c-Myc degradation by inhibiting AKT activation, reduce c-Myc-mediated ASCT2 transcription, ASCT2 catabolises glutamine to supply tumor tissues and promote their progression, ultimately inhibit tumor metastasis by inhibiting glutamine transport [44].

### S-adenosylmethionine metabolism-signaling pathways in CCA

Methionine adenosyltransferase (MAT) produces *S*-adenosylmethionine (SAM), which serves as a biogenic methyl donor for many intracellular biological reactions and is often expressed as MAT1A and MAT2A in mammals [45]. MAT1A is primarily expressed in normal hepatocytes and encodes methionine adenosyltransferase α1 (MATα1). High methylation of the MAT1A promoter has been observed in cholangiocarcinoma. In CCA, MATα1 expression is reduced, while transcription factors such as c-Myc and MafG are highly expressed, and crosstalk between MATα1 and c-Myc/Maf proteins at the promoter level has been identified. Restoration of MAT1A expression or reduction in the expression of transcription factors such as MafG can inhibit CCA growth in vivo, thereby establishing MATα1 as a tumor suppressor gene in CCA [46]. A study using metabolomics sequencing found that the mRNA abundance of key genes involved in methionine metabolism was significantly reduced in HCC and CCA [47].

Integrative histological analysis of iCCA revealed that aberrant RNA editing of the target gene Kip1 ubiquitination-promoting complex 1 (KPC1) is mediated by adenosine deaminases that act on RNA 1A (ADAR1). This ultimately led to the replacement of methionine with valine at residue 8 (p.M8V). Further exploration of the mechanism revealed that KPC1 p.M8V increases NF-κB activity by suppressing the ubiquitination and proteasomal processing of p105 to p50, suggesting that the ADAR1‒KPC1‒NF-κB axis is a potential therapeutic target for iCCA [48]. Similarly, another study revealed that four stemness-associated genes (SDHAF2, MRPS34, MRPL11, and COX8A) that were significantly upregulated in iCCA tissues resulted in negative clinical outcomes. Specifically, these four genes function as stemness-promoting factors by activating the methionine cycle, affecting iCCA cell self-renewal and promoting chemoresistance [49]. *N*-methyltransferase (NNMT) is a member of the *N*-methyltransferase family and plays an important role in tumorigenesis. Significantly elevated levels of NNMT expression were detected in iCCA tissues and correlated with the proliferation and metastasis of iCCA. Mechanistically, NNMT depletes the methyl donor SAM, which leads to the inhibition of histone methylation in iCCA cells and further activates glycolysis in cancer cells through the EGFR-STAT3 signaling pathway [50]. A methionine aminopeptidase-2 (MetAP2) inhibitor may exhibit anti-proliferative and anti-invasive abilities in CCA cell lines through the c-Myc, MMP2, and MMP9 pathways [51].

Methionine adenosyltransferase 1A (MAT1A) is an oncogene whose expression is downregulated in HCC and CCA. Previous studies have shown that the ratio of the expression of MAT1A, an oncogene, to that of MAT2A is positively correlated with HCC survival, the conversion of MAT1A to MAT2A reduces the biosynthesis of S-adenosylmethionine (SAMe), providing favorable conditions for cancer cell growth [52]. There was also a negative correlation between MAT1A and MAFG [46]. A recent study demonstrated that MAFG is upregulated in both HCC and CCA and that MAFG positively regulates MAT2A and c-Myc and negatively regulates MAT1A. Moreover, the induction of MAT2A, an oncogene, also contributes to the increase in MAFG expression. The above mechanism of action may be related to the mutation of the binding sites of four key elements of the human MAFG promoter (E-box, AP-1, NF-κB, and FXR), the inhibitory effect of MAT1A on the promoter activity driven by AP-1, NF-κB, and the E-box, and the inhibitory effect on the expression of c-Myc. The results of the present study suggest that the roles of MAT1A and MAT2A in HCC and CCA are completely opposite, and the specific mechanism of this mechanism still needs to be further investigated via basic experiments [53]. Studies have shown that a direct interaction between MATα1 and 14-3-3ζ occurs and is enhanced by AKT2 phosphorylation of MATα1, and the overexpression of 14-3-3ζ decreases nuclear MATα1 levels and promotes tumor progression; thus, mutual negative regulation of MATα1 and 14-3-3ζ is a key mechanism in liver tumorigenesis [54].

### Serine-threonine metabolism-signaling pathways in cholangiocarcinoma

Metabolomic studies of CCA tissues and normal tissues have revealed active oxidative stress in CCA tissues and high levels of the amino acids serine and glycine, which support tumorigenesis. Phosphoglycerate dehydrogenase (PHGDH), a rate-limiting enzyme in the serine‒glycine pathway, is upregulated in human iCCA. Relatedly, signaling pathway analysis revealed that elevated expression of PHGDH in tumors results in a greater flow of glucose to serine synthesis, promoting metabolic reprogramming of glucose and amino acids, a finding that makes PHGDH a potential therapeutic target for iCCA [55]. In another experiment using CRISPR screening technology, a synergistic anticancer effect of ARV825, a bromodomain and extraterminal domain (BET) PROTAC degrader, was observed when ARV825 was combined with genetic ablation of mTOR pathway members. The mechanism may be due to the synergistic inhibition of PHGDH and phosphoserine aminotransferase 1 (PSAT1), key enzymes of the serine-glycine one-carbon unit metabolic pathway (SGOC), which ultimately leads to epigenetic inhibition of metabolism. These findings also suggest that epigenetic regulation may be a key therapeutic target for further exploration of CCA [56].

Further exploration of the signaling pathways and related targets of serine metabolism reprogramming from a mechanistic perspective reveals that KRAS-driven PHGDH expression mutations alter the reprogramming of serine metabolism in cholangiocarcinoma (CCA), activating the tumor serine-glycine pathway and increasing glucose flux into serine synthesis, ultimately enhancing CCA cell viability [55]. Experiments by Xu et al. [57] clearly demonstrated that SIRT2/c-MYC provides antioxidants to tumor cells, reduces intracellular mitochondrial oxidative phosphorylation levels, increases glucose-to-serine conversion, and ultimately promotes the proliferation of CCA cells via the downstream targets PHDA1 and serine synthesis pathways. SIRT2/c-Myc-induced metabolic reprogramming may represent a novel therapeutic target for the treatment of CCA. The role of cyclic RNAs in amino acid metabolism in CCA has been extensively studied. One study demonstrated that circPCNXL2 directly binds to serine-threonine kinase receptor-associated protein (STRAP) and induces the interaction between STRAP and MEK1/2, thereby promoting iCCA tumor progression through activation of the ERK/MAPK pathway [58]. 14-3-3 is a ubiquitous junction protein in organisms that inhibits the activity of phosphorylated YAP1 by sequestering it in the cytoplasm. In CCA, K50 acetylation (K50ac) of 14-3-3ε leads to activation of YAP1, and the results revealed that 14-3-3ε binds to phosphorylated serine/threonine motifs and may serve as a target for CCA therapy [59].

### Other amino acids metabolism-signaling pathways in CCA

Arginine is a nonessential amino acid in humans. Argininosuccinate synthase acts as a tumor suppressor, converting citrulline to arginine. Defects in argininosuccinate synthase can be observed in CCA and hepatocellular carcinoma (HCC), and a decrease in arginine content is correlated with a decrease in tumor cell proliferation [60, 61]. Arginine can be degraded by a variety of enzymes, including arginine deiminase (ADI) [62]. On this basis, Lynn et al. [60] investigated arginine-depleting polyethylene glycolated arginine deiminase (ADI-PEG20) as a novel anticancer enzyme, which has been shown to be useful in phase I-II trials but needs to be further demonstrated in phase III clinical trials. 5-Hydroxytryptamine, a biogenic monoamine produced from the essential amino acid tryptophan, has a stimulatory effect on cancer cell proliferation, invasion, dissemination, and tumor angiogenesis, interacting with specific receptor subtypes [63]. Higher levels of microcystin-leucine-arginine promote the survival of intrahepatic CCA cells by regulating SET (an mRNA), leading to a poorer prognosis [64]. In further studies, MC-LR was proposed to promote cell proliferation and CCA progression through the Wnt/β-catenin pathway [65]. The above studies suggest that arginine regulates the development of CCA by regulating gene expression, cell invasion and migration, and angiogenesis in CCA. This may represent another signaling pathway associated with amino acid metabolism reprogramming.

An analysis of clinical samples from CCA patients revealed that patients with CCA have increased serotonin in the bile, along with increased expression of tryptophan hydroxylase, which is responsible for the synthesis of 5-hydroxytryptamine, and decreased expression of monoamine oxidase A, which is responsible for the degradation of 5-hydroxytryptamine. Human CCA cell lines also express all 5-hydroxytryptamine receptor subtypes. 5-Hydroxytryptamine administration promoted CCA cell growth both in vivo and in vitro, whereas the inhibition of 5-hydroxytryptamine synthesis suppressed CCA cell growth both in vitro and in vivo. This may serve as a basis for serotonin dysregulation in the bile of CCA patients and make the process of serotonin synthesis an important target for the treatment of CCA [66].

### Mammalian target of rapamycin (mTOR) pathway in CCA

The mTOR complex, another regulator of cell growth and metabolism, is an atypical serine/threonine protein kinase that forms two distinct complexes: mTORC1 and mTORC2 [67, 68]. Activation of the mTOR-related signaling pathway (PI_3_K/AKT/mTOR) was found in both iCCA and eCCA, and its activation correlated with CCA progression and differentiation and reduced OS [69, 70].

The activation of mTORC1 is largely dependent on growth factors and nutrients. Nutrients promote the activation of mTORC1 via phosphatidylinositol 3-kinase (PI_3_K)/AKT signaling. In cellular anabolism, mTORC1 promotes the expression of metabolic genes, especially those related to gluconeogenesis and nucleotide metabolism [71, 72]. Many previous studies have shown that amino acids are associated with mTORC1 activation, as evidenced by the increase in amino acids in cells leading to mTORC1 activation [73]. In CCA, elevated expression of c-Myc induces the expression of amino acid transporter proteins, leading to increased amino acid uptake and mTORC1 activation in HCC cells. However, it has also been suggested that full activation of mTORC1 requires additional signaling induced by amino acids and that the specific mechanism may be related to the transport of L-glutamine and leucine in the cell [74]. In addition, glutamine synthetase (GS), which is downstream of the Wnt/β-catenin pathway, promotes mTORC1 activation in normal liver and HCC, highlighting the important oncogenic role of the Wnt/β-catenin pathway and the mTORC1 signaling cascade in HCC [75].

In animal models with AKT/YAP overexpression, the inhibition of mTOR significantly inhibited iCCA cell growth, and exploration of the mechanism revealed that activated AKT synergized with YAP to support the tumorigenic role of the mTORC2/AKT axis in iCCA [76]. mTORC2 acts directly downstream of the insulin/PI3K cascade; promotes cell survival and proliferation through the activation of AKT and protein kinase C (PKC) kinases; and simultaneously activates mTORC1, creating a positive feedback loop to induce cell growth, metabolism and survival [69]. Moreover, phosphorylated AKT has become a biomarker for mTORC2 activation [77], and the mechanism that triggers mTORC2 activation in CCA and HCC is not yet known; notably, a few studies indicate that it is related to copy number gain of RICTOR [78].

Overall, mTOR is closely associated with protein metabolism, amino acid uptake and energy stress in cancer cells. Baicalein is a bioactive constituent of *Scutellaria baicalensis Georgi*, and a study by Li demonstrated that baicalein inhibits the mTOR signaling pathway and induces apoptosis by downregulating glutamine metabolism. Specifically, baicalein interacts with and inhibits the activation of glutamine transporter proteins and glutaminase, suppressing mTOR expression and ultimately altering glutamine metabolites [79]. The idea of altering amino acid metabolism and energy stress in the microenvironment through metabolic reprogramming at the tumor site to inhibit mTOR activation is also emerging as a new research hotspot for CCA therapeutic regimens. The antitumour and immunosuppressive properties of mTOR inhibitors have been demonstrated in many clinical trials [80, 81].

### Metabolism-immunity axis

A study revealed the metabolic subtypes of intrahepatic cholangiocarcinoma (iCCA) through multi-omics analysis and identified diacylglycerol kinase α (DGKA) as a potential therapeutic target. The study mentioned that different metabolic subtypes of iCCA have significant differences in metabolic and immune characteristics, with some metabolic genes co-enriched with the expression of T cell exhaustion markers (such as PD-1 and LAG-3) and M2 polarization markers (such as CD163 and ARG1). This result suggests that there are certain links between different types of amino acid metabolism reprogramming and the tumor immune microenvironment in cholangiocarcinoma, which is the “metabolism-immunity” axis mentioned later. Here, we summarize the possible metabolism-immunity axis, hoping to find potential therapeutic targets by clarifying the relationship between amino acid metabolism and the immune microenvironment. Studies have shown that upregulation of GLS1 expression in CCA cells enhances glutaminolysis, thereby altering the acidic environment of the tumor microenvironment, increasing stromal alkalinization, and stimulating the polarization of tumor-infiltrating macrophages to the M2 type [82]. In addition to glutamine, a study on human CCA tissues found that arginase ARG1 was highly expressed and positively correlated with M2 macrophage infiltration, indicating that arginine metabolism reprogramming also affects the polarization of tumor-associated macrophages. Local arginine depletion also inhibits T cell mTORC1 expression, synergistically upregulates PD-1, and mediates the immune evasion of CCA cells [83]. In addition to macrophages, cancer-associated fibroblasts (CAFs) secrete glutamine to maintain CCA cell mTORC1 activity and induce the stabilization of HIF-1α in CAFs themselves, forming a positive feedback metabolic loop [84]. Similarly, CAFs highly express phosphoglycerate dehydrogenase (PHGDH) and export serine to CCA cells. The serine secreted by CAFs may be taken up by other stromal or immune cells, thereby altering the functional state of these cells and further promoting tumor progression [85]. CD8⁺ T cells can secrete a variety of cytokines to regulate other immune cells in the tumor microenvironment. After glutamine deprivation, the lactate levels in CCA supernatant are reduced, which is conducive to the recovery of CD8⁺ T cells’ ability to secrete IFN-γ, forming a positive immune activation loop to inhibit CCA [86]. In addition, the IDO1 inhibitor epacadostat was also found to restore the function of CD8⁺ T cells in organoid-T cell co-culture models [87]. Studies have shown that elevated serum kynurenine/tryptophan ratios are associated with early recurrence after surgery in iCCA patients. This may be related to the overactivation of the IDO1/TDO2 pathway, thereby exacerbating the immunosuppressive microenvironment [88]. Kynurenine also promotes the proliferation of Treg cells in CCA through the AhR pathway to promote the immunosuppressive microenvironment [89]. The aforementioned findings indicate that amino acid metabolism reprogramming affects the tumor immune microenvironment through a variety of mechanisms, including the regulation of immune cell polarization, function, and metabolic status. Targeting these metabolic pathways (such as GLS1, ARG1, PHGDH, IDO1, etc.) may restore the function of immune cells, inhibit tumor progression, and provide new strategies for the treatment of cholangiocarcinoma.

**Fig. 2:**
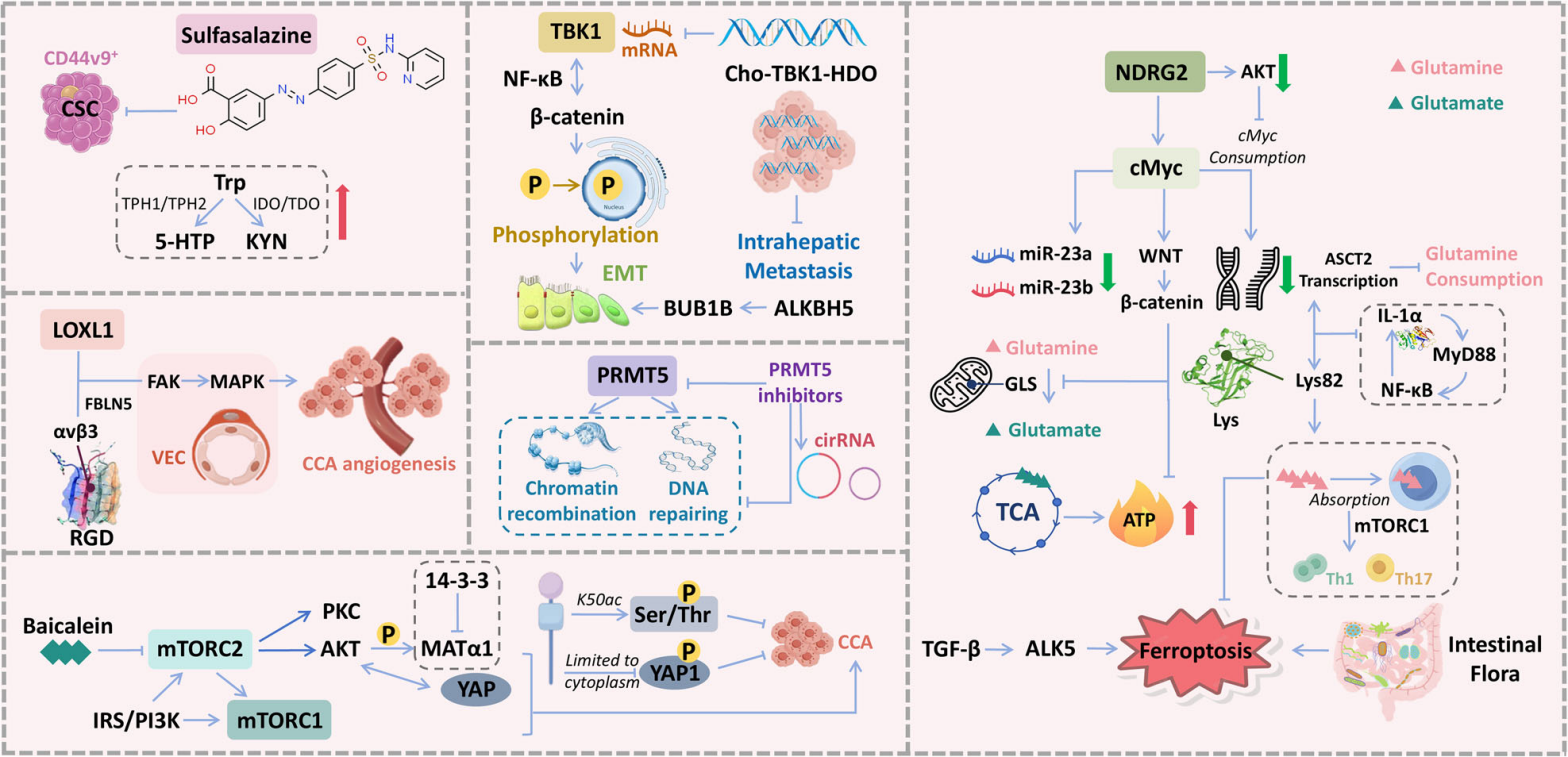
Integrated signaling pathways in cholangiocarcinoma (CCA). Sulfasalazine, a drug targeting CD44v9^+^ cancer stem cells (CSCs), influences tryptophan metabolism through enzymes TPH-1/TPH2, leading to the production of 5-HTP and KYN. TBK1 activation suppresses NF-κB signaling and β-catenin phosphorylation, which are crucial for epithelial-mesenchymal transition (EMT) and intrahepatic metastasis in CCA. Cho-TBK1-HDO represents a potential inhibitor of this pathway. LOXL1 promotes CCA angiogenesis by interacting with fibronectin (FBLN5) and integrin αvβ3 on vascular endothelial cells (VECs) through the FAK-MAPK signaling pathway. PRMT5 is involved in chromatin recombination and DNA repair. Its activity can be modulated by inhibitors and circular RNAs (circRNAs), indicating a potential therapeutic target. The mTOR signaling pathway includes mTORC1 and mTORC2 complexes, which regulate cell growth and survival through IRS/PI3K, PKC, AKT, and other downstream effectors. Baicalein, a natural compound, inhibits mTORC2, affecting YAP and MATα1, thereby limiting CCA progression. Indoleamine 2,3-dioxygenase (IDO) and tryptophan 2,3-dioxygenase (TDO) are enzymes involved in tryptophan catabolism, potentially influencing the tumor microenvironment. BUB1B and ALKBH5 are implicated in cell cycle regulation and DNA repair mechanisms, which are essential for maintaining genomic stability in cancer cells. NDRG2 activation leads to a decrease in AKT, which is negatively regulated by c-Myc, affecting cellular metabolism and survival. c-Myc promotes the transcription of ASCPT2, which is involved in glutamine, glutamate, and glucose metabolism. It also influences β-catenin via the WNT signaling pathway, impacting cell proliferation and survival. miR-23a and miR-23b negatively regulate GLS, affecting the tricarboxylic acid (TCA) cycle and ATP production. ASCPT2 transcription is induced by c-Myc and is involved in lysine metabolism. Lysine can be converted to Lys82, which interacts with IL-1α and MyD88, influencing NF-κB signaling. The mTORC1 complex is involved in nutrient sensing and cell growth regulation. It is influenced by the intestinal flora and ferroptosis, a form of regulated cell death. The intestinal flora can influence the development of CCA through the promotion of ferroptosis, which is regulated by TGF-β and ALK5 signaling. The interaction between CCA and immune cells, including Th1 and Th17 cells, which can influence tumor progression and immune evasion. CSC cancer stem cell, VEC vascular endothelial cell, Trp tryptophan, 5-HTTP: 5-hydroxytryptophan, KYN kynurenine, TPH-1 Trp hydroxylase 1 enzyme, TPH2 Trp hydroxylase 2 enzyme, IDO indoleamine 2, 3-dioxygenase, TDO tryptophan 2,3-dioxygenase, EMT epithelial-mesenchymal transition, BUB1B BUB1 mitotic checkpoint serine/threonine kinase gene, MetAP2 methionine aminopeptidase-2, Baicalein 5,6,7-trihydroxyflavone, RGD Arg-Gly-Asp, YAP yes-associated protein, TBK1 TANK-binding kinase 1, ALKBH5 Alk B homolog 5, PRMT5 protein arginase methyltransferase 5, mTOR mammalian target of rapamycin, LOXL1 lysyl oxidase-like 1, Ser serine, Thr threonine, Cho-TBK1-HDO cholesterol-conjugated DNA/RNA heteroduplex oligonucleotides targeting TBK1. NDRG2 NDRG family member 2 gene, GLS glutaminase, TCA tricarboxylic acid cycle, ALK5 activin receptor-like kinase 5, mTOR mammalian target of rapamycin, ASCT2 glutamine transporter carrier 2, MyD88 myeloid differentiation factor 88, Lys lysine.

Overall, signal transduction pathways in cholangiocarcinoma (CCA) are intricately intertwined. The activation of TBK1 suppresses NF-κB signaling and β-catenin phosphorylation, which are critical for epithelial-mesenchymal transition (EMT) and intrahepatic metastasis. Amino acids such as tryptophan, serine, and threonine affect CCA progression through the PI3K/AKT/mTOR pathway. The mTOR signaling pathway, comprising mTORC1 and mTORC2 complexes, regulates cell growth and survival through downstream effectors such as IRS/PI3K, PKC, and AKT. PRMT5 is involved in chromatin remodeling and DNA repair, with its activity modulated by inhibitors and circular RNAs (circRNAs). The mTORC1 complex is implicated in nutrient sensing and cell growth regulation, influenced by the gut microbiota and ferroptosis, a form of regulated cell death. As a transcription factor, c-Myc affects glutamine metabolism mainly through the c-Myc/WNT/Wet pathway and is closely related to cancer cell glucose metabolism. Additionally, the c-Myc-regulated transcription of ASCPT2 influences cellular metabolism and survival via the WNT signaling pathway. It also affects cancer cell apoptosis and iron death by influencing the expression of tumor immune cells and has an important role in hypoxia-induced chemoresistance. The interaction between CCA and immune cells, including Th1 and Th17 cells, impacts tumor progression and immune evasion (Fig. 2). The induction of key proteins or key signaling pathways corresponding to different amino acids in CCA can help to further understand the role of amino acid metabolism in cholangiocarcinoma (Table 2).

**Table 2:** Key proteins/signaling pathways related to the metabolism of different amino acids in CCA.

Amino acids	Key proteins/signaling pathways	Experimental content	Experimental conclusions	Ref.
Glutamine	miR-23amiR-23b	Investigation of glutamine metabolism in cholangiocarcinoma (CCA), focusing on the regulation by c-Myc.	c-Myc inhibits miR-23a and miR-23b, upregulating mitochondrial glutaminase to enhance glutamine catabolism and ATP production.	[41]
	NDRG2 c-Myc/WNT/Wet	Study of the effects of NDRG2 on glutamine metabolism via the c-Myc/WNT/Wet-catenin pathway.	NDRG2 inhibits Akt activation, reducing c-Myc-mediated ASCT2 transcription and suppressing glutamine uptake and tumor metastasis.	[137]
	GLS,GS	Examination of the role of glutamine metabolism in chemotherapy resistance in CCA.	Glutamine metabolism is associated with chemoresistance; low glutamine levels increase cellular sensitivity to apoptosis.	[34]
		Investigation of the effects of traditional Chinese medicine extracts on glutamine metabolism in CCA.	Aloperine (a berberine extract) inhibits glutamine hydrolysis, reducing glutamate levels and suppressing the growth of IDH-mutant CCA cells.	[101]
Tyrosine	IDO1 TDO2 kynurenine	Study of tryptophan metabolism in CCA, focusing on the role of IDO1 and TDO2 in immune evasion.	Tryptophan metabolites (e.g., kynurenine) inhibit T-cell activity, promoting immune evasion; IDO1/TDO2 are potential targets for immunotherapy.	[99]
	FGFR2 PTPN9	Investigation of the effects of tyrosine kinase inhibitors in CCA, focusing on FGFR2 fusion mutations.	FGFR2 kinase inhibitors (e.g., Pemigatinib) suppress CCA cell proliferation and metastasis by inhibiting FGFR2 phosphorylation.	[108]
5-hydroxytryptamine	TPH-1 5-HT receptors	Examination of the role of 5-hydroxytryptamine (serotonin) metabolism in CCA, focusing on TPH-1 expression.	Elevated serotonin synthesis is associated with CCA proliferation and chemoresistance; inhibiting serotonin synthesis enhances chemotherapy efficacy.	[97]
Methionine	PI3K/AKT/mTOR mTORC1 mTORC2	Examination of the role of the mTOR signaling pathway in CCA, focusing on amino acid metabolism and mTORC1 activation.	Amino acid metabolism activates mTORC1 via the PI3K/AKT pathway, promoting CCA cell growth and metabolic reprogramming.	[69, 70]

This table summarizes the experimental studies focusing on the role of various proteins and signaling pathways in the metabolism of amino acids in CCA. It includes the amino acids involved, key proteins or pathways, experimental content, experimental conclusions, and references.

## Therapies targeting amino acid metabolism

### Amino acid metabolism-related treatments

Therapies targeting tumor metabolism have been investigated in basic experiments. In hepatocellular carcinoma (HCC) and cholangiocarcinoma (CCA), MAT1A expression is reduced, and the induction of FOXM1 and NF-κB expression is accompanied by a decrease in MATα1 (the protein encoded by MAT1A) expression, while MATα1 positively regulates the expression of p50 and p65. Moreover, the binding of MATα1 blocks the FOXM1 promoter and binds and inhibits NF-κB elements in the presence of p65 or p50 [90]. Another study showed that the histone lysine acetyltransferase KAT2B interacts with SP1 to regulate NF2–YAP signaling and inhibit CCA growth; thus, this signaling cascade may be a target for CCA treatment [91]. A multicentre randomized phase II study enrolling 105 patients demonstrated that nanvuranlat, an L-type amino acid transporter protein 1 inhibitor, improved the progression-free survival (PFS) of patients with advanced and refractory biliary tract cancers and presented a reliable biosafety profile; thus, this treatment that warrants further study in currently untreatable CCA patients [92]. The use of L-asparaginase as a first-line treatment option in nonsolid tumors results in nutrient-deficient tumor cells, thereby limiting tumor progression [93]. CDC-like kinase 3 (CLK3) functions on serine/threonine- and tyrosine-containing substrates to directly phosphorylate USP13 and promote its binding to c-Myc. In turn, c-Myc transcriptionally upregulates CLK3, forming a positive feedback loop. These findings suggest that CLK3 influences metabolic reprogramming and has potential therapeutic utility [94].

Although some drugs demonstrate efficacy in early clinical trials, they may encounter setbacks in subsequent studies. For instance, the Phase II trial combining ADI-PEG-20 with the mFOLFOX6 chemotherapy regimen for hepatocellular carcinoma failed to show a significant therapeutic advantage. This may be attributed to tumor heterogeneity, complex metabolic pathways, and inherent limitations of the drug itself [95]. Regarding drug development, the primary challenge is overcoming poor drug stability. Some amino acid-based drugs are rapidly cleared from the body, resulting in insufficient concentrations to exert therapeutic effects. Additionally, certain drugs lack target specificity, potentially affecting multiple targets simultaneously, which may lead to adverse reactions or diminished efficacy. Regarding clinical trial design, multiple factors must be considered, such as patient tumor characteristics, genetic background, and sample size. Failure to adequately account for these factors may lead to suboptimal trial outcomes. Currently conducted clinical trials universally suffer from insufficient sample sizes, potentially preventing accurate reflection of a drug’s true efficacy and safety. Future multicenter, prospective studies represent the current strategy to address these challenges. Although numerous biomarkers related to amino acid metabolism have been identified in basic research, their clinical application remains challenging. Further validation is required to establish their accuracy and reliability across diverse patient populations. Additionally, due to the heterogeneity among cholangiocarcinoma cells, different cell types exhibit varying degrees of dependence on amino acid metabolism. This heterogeneity makes it difficult for drugs targeting a single amino acid metabolic pathway to effectively inhibit all tumor cells. Finally, the tumor microenvironment of cholangiocarcinoma may harbor immunosuppressive factors that compromise the efficacy of amino acid metabolism inhibitors. As previously analyzed, certain amino acid metabolites may participate in regulating immune cell function. While this opens possibilities for novel immunotherapy approaches, it may also diminish therapeutic outcomes due to alterations in the immune microenvironment.

### Amino acid metabolism and chemoresistance

In addition to providing new therapeutic targets and research directions, studies on amino acids have revealed their involvement in the side effects of chemotherapy in patients with CCA. Katsuya Nagaoka et al. [96] demonstrated that elevated 2-oxoglutarate (2-OG) antagonizes the DNA damage response (DDR) in CCA patients treated with chemotherapy by modulating aspartate β-hydroxylase. Specifically, elevated 2-OG and full-length aspartate β-hydroxylase (ASPH) antagonize the effects of the DDR on chemoresistance. Targeting ASPH may increase the DDR and improve chemotherapy efficacy in CCA patients. 5-hydroxytryptamine may also attenuate chemoresistance in tumor cells. Increased serotonin accumulation and secretion are associated with CCA cell tumorigenecity [66], and patients with high TPH-1 expression and low serum 5-hydroxytryptamine expression have better long-term outcomes [97]. On the basis of these findings, telotristat ethyl, a tryptophan hydroxylase inhibitor that blocks serotonin biosynthesis, was combined with standard chemotherapy in another study, and the results demonstrated a cumulative effect of tumor suppression, especially in patients with serotonin overexpression. Furthermore, the inhibition of 5-hydroxytryptamine increased the sensitivity of CCA cells to current chemotherapies [98]. An analysis of clinical samples from CCA patients revealed that the number of CD44v9-positive cells was significantly greater in CCA patients than in normal individuals. Salazosulphapyridine (SSZ) works by inhibiting the proliferation of CD44v9-expressing cancer stem-like cells (CSCs). In this process, tryptophan degradation pathways (the kynurenine metabolism pathway and 5-hydroxytryptamine pathway) are activated, which increases the sensitivity of tumor cells to CIS; therefore, SSZ combined with CIS has a greater tumor cell inhibitory effect than SSZ alone [99].

Targeting glutamine metabolism in CCA can also serve as a synergistic approach to current chemotherapy regimens. Compared with those in normal tissues, glutamine levels are decreased in CCA tissues, and c-Myc expression is reduced in glutamine-deficient cell lines; in contrast, the combination of cisplatin (CIS) and hypoxia leads to sustained c-Myc protein expression in cells. It is therefore reasonable to assume that the regulation of glutamine metabolism is a key factor in overcoming resistance in CIS-resistant SCK (SCK-R) cells [34]. Targeting the LAT2/glutamine pathway can inhibit cell proliferation by reducing the expression of glutamine deaminase and glutamine synthetase, thereby enhancing the sensitivity of CCA cells to gemcitabine. This provides an alternative treatment option for CCA patients resistant to gemcitabine [100]. Additionally, inhibiting the hydrolysis of glutamine to glutamate can reduce D-2-hydroxyglutarate (D-2HG) levels, thereby overcoming chemoresistance caused by direct targeting of mutant isocitrate dehydrogenase (IDH) [101]. These findings suggest that glutamine metabolism is closely related to chemoresistance in CCA cells. In another study, a glutamine-deficient (GD) CCA cell line was constructed, and GD cells were treated with CIS or gemcitabine under normoxic and hypoxic conditions. The results revealed that the resistance of CCA cells to chemotherapeutic drugs was reversed under hypoxic conditions, and a decrease in c-Myc expression was observed in GD cells, indicating that c-Myc has an important function in hypoxia-induced chemotherapeutic drug resistance [30]. Overall, glutamine plays a pivotal role in the energy metabolism and growth proliferation of cholangiocarcinoma cells. Its metabolic reprogramming is closely associated with chemotherapeutic resistance. Inhibition of glutamine metabolism or combined inhibition of glutamine and glucose uptake can effectively reverse chemotherapeutic resistance, thereby providing a novel therapeutic strategy for cholangiocarcinoma.

### Immunotherapy and amino acid metabolism

The development of immunotherapy for CCA is dependent on the identification of immune checkpoints that can be inhibited and the understanding of tumor immunogenicity. Additionally, in the field of immunotherapy, Mussarat Wahid et al. [102] found that serine-arginine-rich splicing factor (SRSF1) regulates the selective splicing of PD-1, resulting in the selective splicing of PD-1 and the formation of soluble antagonist isoforms that are endogenously expressed in T cells, preventing the depletion of T cells by tumors. Arginase is secreted by myeloid suppressor cells in the tumor microenvironment and is a major regulator of arginine-mediated immune responses. A phase I/II study of the arginase inhibitor INCB001158 in combination with current first-line chemotherapy regimens was performed to evaluate the safety, efficacy, and synergistic effects of this combination in patients with advanced biliary tract cancer. Data from the study revealed that some patients with advanced CCA benefited. Further exploration of the mechanism of action revealed that INCB001158, an arginase inhibitor, increases the concentration of arginine in the tumor microenvironment, reverses the immunosuppressive effects of neutrophils and myeloid-derived suppressor cells on T cells, and activates the normal cellular immune process of the body, which ultimately inhibits tumor growth [103]. A study revealed that the tyrosine motif structural domain of the immunoreceptor (TIGIT) is upregulated during immune T lymphocyte depletion in patients with CCA and that TIGIT recognizes the differentiating depleted T lymphocytes better than PD-1 did. These findings suggest that TIGIT-targeted therapy may be a new therapeutic strategy for CCA patients [104].

### Targeted therapy and amino acid metabolism

FGFR, an important molecule in the signaling pathway, is responsible for the regulation of cell proliferation and angiogenesis, and the most common types of variants in CCA are FGFR2 fusions and mutations, which makes drugs targeting FGFR variants highly valuable in the treatment of CCA [105]. Pemigatinib targets and inhibits FGFR2 phosphorylation and has become a first-line drug for CCA-targeted therapy [106]. A phase II clinical trial demonstrated the efficacy of pemigatinib and suggested that it may be a second-line treatment option for patients who have progressed after platinum-based regimens [107]. Zhao et al. [108] found that the protein tyrosine phosphatase PTPN9 interacts with FGFR2 to synergistically increase the efficacy of pemigatinib and inhibit CCA cell proliferation, migration and invasion by inhibiting FGFR2 Y656/657 phosphorylation. Caitlin B Conboy et al. [109] found that a novel LCK-selective TKI (NTRC 0652-0) effectively inhibited YAP tyrosine phosphorylation and cotranscriptional activity and was well tolerated and cytotoxic in a variety of preclinical models, and that this approach may be effective in YAP-dependent or FGFR2-fused CCAs, which is currently under investigation in a clinical phase I trial. FGFR-specific tyrosine kinase inhibitors (F-TKIs) provide clinical benefit in iCCA, but FGFR2 fusion proteins carrying mutations in key tumor-associated sites confer a growth advantage to tumor cells and increase resistance to selective TKIs. Studies have shown that lenvatinib is a promising therapeutic option for FGFR2-driven CCA when insurmountable selective TKI occurs [110].

CCAs exhibit diverse mutational profiles, with ~4–5% having the B-Raf proto-oncogene serine/threonine kinase (BRAF) V600E mutation [111]. A recent phase II multicentre clinical trial in patients with the BRAF V600E mutation investigated the efficacy and safety of the combination of dabrafenib and trametinib in these patients, with promising results [112]. Although HER2 mutations are rare in CCA, neratinib, a tyrosine kinase inhibitor that targets HER, has demonstrated antitumour activity in the treatment of patients with HER-mutated CCA. Mutations in HER2, a receptor tyrosine kinase, are rare genomic events in biliary tract cancer (BTC). Neratinib, an irreversible pan-HER oral TKI, has shown antitumour activity in patients with HER2-mutated refractory BTC. The mechanism may be related to interference with the activation of constitutive receptor kinases [113]. In addition, zongertinib (BI 1810631), another HER2 inhibitor, has been shown to inhibit the growth of cells in a manner dependent on HER2 oncogenic driver events [114].

Heterozygous mutations in the catalytic arginine residues (IDH1 and IDH2) of isocitrate dehydrogenases 1 and 2 are common in CCA. Mutations in IDH are predominantly observed in iCCA and are associated with poor differentiation of hepatic progenitor cells due to production of the cancer metabolite D-2-HG [115]. A multicentre, randomized, controlled, phase III clinical study of 230 patients revealed a significant improvement in progression-free survival in patients treated with ivosidenib, a small-molecule targeted inhibitor of mutant IDH1, suggesting that targeting IDH1 mutations in advanced IDH1-mutant CCA could have a significant clinical benefit [116]. Another multicentre study also demonstrated the clinical benefit of oral small-molecule inhibitors of mutant IDH1 in patients with advanced IDH1-mutant CCA [117]. Another study in nonsolid tumors demonstrated that mutant isocitrate dehydrogenase 1 (mIDH1) drives tumorigenesis through the production of the oncometabolite R-2-hydroxyglutarate (R-2-HG) in a variety of nonsolid tumors and that the use of mIDH1 inhibitors appeared to have a significant inhibitory effect on hematological tumors [118]. Since Src (a protein tyrosine kinase) modifies the membrane-associated guanylate kinase, WW, and 1-containing PDZ structural domain (MAGI1) in the mIDH1 CCA, mIDH1/2 cancers are dependent on Src kinase signaling and sensitive to Src inhibition [119]. Studies have shown that Src inhibition can restore the tumor suppressor function of MAGI1-PP2A, such as the S6K/AKT inhibitor M2698, suggesting the therapeutic value of Src inhibitors in mIDH CCA [120]. Similarly, a phase II trial using dasatinib, a multikinase inhibitor, for the treatment of mIDH CCA is ongoing (NCT02428855).

### Immune microenvironment and amino acid metabolism

The functional plasticity of immune cells is closely related to metabolic reprogramming, mainly manifested as metabolic reprogramming providing energy and substrates for the activation of immune cells [121]. For instance, Teffectors (Teff) exert their effects after T-cell receptors are activated by antigens and co-stimulated through MTOR-dependent processes, including aerobic glycolysis (Warburg-type metabolism). Activated Teff cells undergo cell division to convert glucose and glutamine into energy substances. This process requires the uptake of a large amount of amino acids and depends on the TORC1, Pl3K, and AKT pathways. Meanwhile, the activation of AKT will prevent the activation of Treg cells, resulting in defects in the quantity and function of Treg, and further enhancing the killing effect of T cells on tumor cells. Other metabolic pathways that affect the function of Treg, such as arginine, also help keep Teff in a more easily activated state. M2 macrophages hydrolyze arginine through ARG1 (arginase 1) and limit the supply of arginine to Teff by consuming the surrounding arginine, thereby inhibiting the activation of Teff [122]. Therefore, altering the amino acid supply at the tumor site can affect the spark action of immune cells, change the immune surveillance of tumor cells, influence tumor growth, metastasis and chemoresistance and may become the direction for the future treatment of CCA. In a study of CCA, Wu et al. [123] reported that mIDH1 inhibits CD8^+^ T-cell recruitment and IFNγ-TET2 signaling and promotes immune escape and tumor maintenance in CCA, demonstrating the importance of immune function and the IFNγ-TET2 axis in mIDH1 inhibition. This study provides a new idea for immunotherapy that restores T-cell immunity through the IFNγ-TET2 axis after mIDH1 inhibition and its potential combination with CTLA4 blockade.

Metabolic rearrangements in glutamine metabolism also influence the immune microenvironment. IDH mutations cause α-ketoglutarate (α-KG) in cells to be converted into D-2-hydroxyglutarate (D-2HG). D-2HG significantly suppresses the proliferation of activated T cells, including various CD4^+^ T cell subsets such as Th1, Th17, and Treg, thereby reducing T cell numbers. It also suppresses the secretion of key cytokines such as IFN-γ by T cells, impairing their immune function [124]. Furthermore, studies demonstrate that D-2HG accumulation promotes the differentiation of FOXP3^+^ CD4^+^ Treg cells to some extent while inhibiting the secretion of inhibitory cytokines like IL-10. This ultimately establishes an “immunologically cold” tumor phenotype, unfavorable for T cell infiltration and immune responses [125]. These findings confirm that amino acid rearrangements caused by IDH mutations contribute to alterations in the tumor immune microenvironment. However, the precise extent of their impact on cholangiocarcinoma cells requires further experimental validation.

### Emerging therapeutic technologies related to amino acids

Moreover, novel nanotherapeutic technologies based on glutamine metabolism have been investigated in CCA. Zheng et al. [126] developed a nanosystem, R-CM@MSN@BC, to load reactive oxygen-responsive diselenide onto silicone nanoparticles to control the activity of the glutamine metabolism inhibitor bis-2-(5-phenylacetamido-1,3,4-thiadiazol-2-yl)ethylsulphide (BPTES), which modulates the necrotic process of tumor cells. One study revealed that remodeling of the immunosuppressive tumor microenvironment by affecting intracellular amino acid metabolites and the combination of this approach with immunotherapy improved the efficacy of PD-L1 immunotherapy. On the basis of the mechanism of arginine in CCA, nanomaterials have been mentioned as a novel research hotspot. Wang et al. [127] successfully developed a comprehensive nanotherapeutic platform called CMArg@Lip, which incorporates L-arginine and combines photodynamic therapy (PDT) and gas therapy with the addition of the NRF2 inhibitor ML385 to construct ROS-responsive liposomes. CMArg@Lip significantly affects immunomodulation and downregulates the expression of PD-L1 in tumor cells. This protein promotes the antitumour function of cytotoxic T lymphocytes by activating the STING signaling pathway in living myeloid-derived suppressor cells, thereby reprogramming the immunosuppressive microenvironment through various mechanisms. However, the biological safety of nanotherapy in clinical settings remains a pressing issue that needs to be addressed [128]. Additionally, improving the bioavailability of nanomaterials through modified preparation methods or synthesis schemes is also a current research objective.

## Discussion

Cholangiocarcinoma (CCA) is a complex and heterogeneous disease, with the intrahepatic and extrahepatic subtypes presenting distinct clinical challenges. Intrahepatic cholangiocarcinoma (iCCA) often has no early clinical manifestations and no clear and effective biomarkers, which leads to late diagnosis of CCA and also to limited treatment options [129]. Currently, the standard chemotherapy regimen for iCCA is a combination of gemcitabine and cisplatin, and systemic therapies, including targeted therapies and immunotherapies, have progressed in recent years; however, the prognosis for CCA patients remains poor [16, 130]. These findings highlight the urgent need for novel therapeutic strategies that address the underlying metabolic and genetic drivers of this disease. Recent studies have emphasized the role of amino acid metabolism in the progression of CCA. Glutamine, for example, is a critical nutrient for cancer cell survival and proliferation, and its metabolism is closely linked to the Warburg effect and the ability of cancer cells to thrive in hypoxic environments. Targeting glutamine metabolism has shown promise in overcoming chemoresistance and improving treatment outcomes. Similarly, the dysregulation of other amino acids, such as arginine, tryptophan, and serine, has been implicated in CCA progression, suggesting potential therapeutic avenues. Taken together, these findings suggest that the process of reprogramming amino acid metabolism may be a new target for prolonging survival in patients with advanced CCA.

**Fig. 3:**
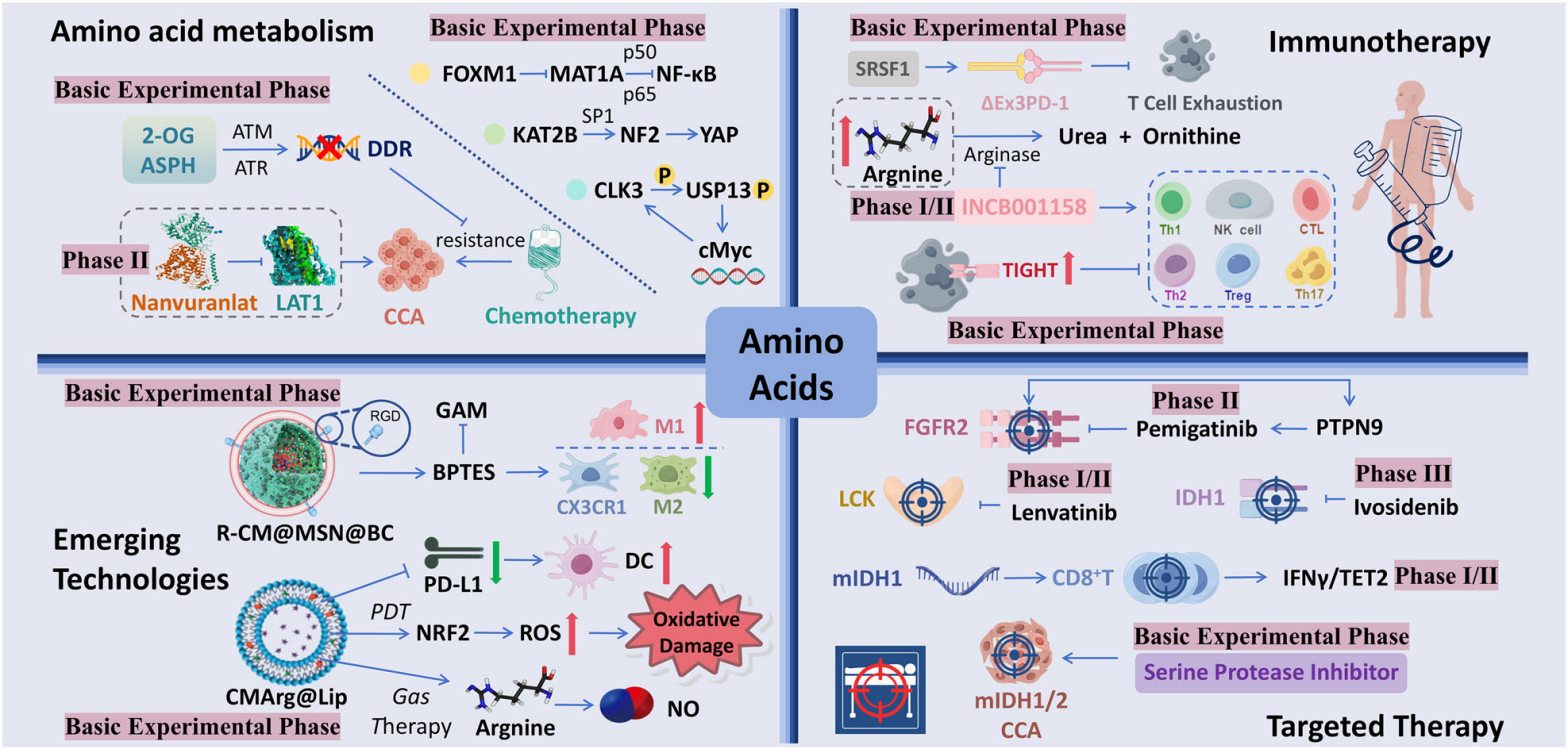
Role of amino acid metabolism in cholangiocarcinoma (CCA) therapy. Key metabolic enzymes like FOXM1-MAT1A, KAT2B-NF2-YAP, and CLK3-USP13 drive CCA progression and chemoresistance. The folate cycle and aspartate metabolism, indicated by 2-OG and ASPH, are involved in CCA. Nanuvuralat and LAT1 target amino acid transport and resistance. Amino acid metabolism impacts T cell exhaustion, with ΔEx3PD-1 as an immunotherapy target. Arginine metabolism, particularly arginase activity, depletes arginine, affecting T cell function and aiding tumor immune evasion. INCB001158, an inhibitor of TIGHT, may boost immune responses against CCA. FGFR2, IDH1, and LCK are molecular targets for therapies like pemigatinib, ivosidenib, and lenvatinib. Serine protease inhibitors also show potential in CCA targeting. The interplay between amino acid metabolism and immune cell function, including Th1, Th2, Treg, NK, and CTL cells, suggests possible immunomodulatory therapies. Additional strategies include gas therapy, PDT, and drug delivery via R-CM@MSN@BC and CMArg@Lip. Oxidative stress and the PD-L1/PD-1 axis in CCA indicate potential immune checkpoint inhibitor targets. LAT1 L-type amino acid transporter 1, 2-OG: 2-oxoglutaric acid; ASPH aspartate β-hydroxylase, ATM ataxia-telangiectasia mutated proteins, ATR ATM and Rad3-related proteins, DDR DNA damage response, FOXM1 forkhead box protein M1, MAT1A methionine adenosyltransferase 1A, KAT2B K (lysine) acetyltransferase 2B, SP1 specificity protein 1, YAP yes-associated protein, CLK3 CDC-like kinase 3, USP13 ubiquitin-specific peptidase 13, c-Myc myelocytomatosis viral oncogene homolog, SRSF1 serine-arginine-rich splicing factor, SRPK1 SR protein kinase 1, TIGHT T cell immunoreceptor with Ig and ITIM domains, GAM glutamine metabolism, RGD Arg-Gly-Asp, PDT photodynamic therapy, NRF2 nuclear factor erythrocyte 2 associated factor 2, PD-L1 programmed death ligand 1, PD-1 programmed death 1, DC dendritic cells, LCK lymphocyte-specific protein tyrosine kinase, mIDH1 IDH1 mutation.

In the therapeutic aspect, amino acids mainly affect the treatment of CCA through metabolic processes and metabolites. In addition, the synergistic effect of amino acid metabolism with immunotherapy and targeted therapies is a hot research topic at present, and the interaction with some new therapeutic strategies, such as bioengineering, has broadened the disciplinary boundaries of future research (Fig. 3). Currently, clinical studies on amino acid metabolism in CCA are mainly focused on targeted and immunological drugs, and there is still much room for development (Table 3). The tumor microenvironment also plays a crucial role in shaping the metabolic landscape of CCA. Immune checkpoint inhibitors and novel nanotherapeutic technologies can modulate the immunosuppressive environment and enhance antitumour immunity. In addition, the relationships between specific gene mutations and metabolic pathways are being explored. Amino acid metabolism, as one of the three major metabolic pathways, and future synergies between immunotherapy, targeted therapy, and novel nanotechnology treatments are powerful punches currently being thrown to overcome the therapeutic challenge of CCA.

**Table 3:** Clinical studies and research involving amino acid metabolism in CCA.

Primary participants	Key proteins	Experimental content	Experimental conclusions	Ref.
Patients with BRAF V600E-mutated CCA	BRAF V600E mutations	Phase II clinical trial of the combination therapy with Dabrafenib and Trametinib in patients harboring BRAF V600E mutations	The combination therapy demonstrated promising outcomes in patients with BRAF V600E-mutated cholangiocarcinoma (CCA)	[112]
Patients with FGFR2 fusion mutations and unresectable CCA	FGFR2 fusion mutations	Investigational study of Infigratinib in patients with FGFR2 fusion mutations and unresectable cholangiocarcinoma (CCA)	Infigratinib exhibited significant therapeutic benefits in patients with FGFR2 fusion mutations and unresectable CCA	[138]
		Phase II clinical trial of Pemigatinib in patients with FGFR2 fusion mutations	Pemigatinib demonstrated efficacy in patients with FGFR2 fusion mutations and could serve as a second-line treatment option	[106, 107]
Patients with advanced and refractory biliary tract cancers	L-type amino acid transporter protein 1	Phase II multicenter clinical trial of Nanvuranlat (an L-type amino acid transporter protein 1 inhibitor) in patients with advanced and refractory biliary tract cancers	Nanvuranlat improved progression-free survival (PFS) in patients with advanced and refractory biliary tract cancers, with a reliable biosafety profile	[92]
Patients with advanced biliary tract cancer	Arginase, INCB001158	Phase I/II study evaluating the safety, efficacy, and synergistic effects of the arginase inhibitor INCB001158 in combination with first-line chemotherapy.	Some patients with advanced CCA benefited. INCB001158 increases arginine levels in the tumor microenvironment, reversing immunosuppression and inhibiting tumor growth.	[103]
Patients with IDH1-mutated CCA	IDH1	Phase III clinical trial of Ivosidenib (a small-molecule IDH1 inhibitor) in patients with advanced IDH1-mutated CCA	Ivosidenib significantly improved progression-free survival in patients with advanced IDH1-mutated CCA	[116]
		Multicenter study of oral small-molecule IDH1 inhibitors in patients with IDH1-mutated CCA	Oral small-molecule IDH1 inhibitors demonstrated clinical benefits in patients with IDH1-mutated CCA	[117]
		Ongoing Phase II clinical trial of Dasatinib (a multikinase inhibitor) in patients with mIDH CCA	Dasatinib showed therapeutic potential in patients with mIDH CCA, with the study still in progress	NCT02428855
	Src MAGI1	Phase II clinical trial of Src inhibitors in patients with mIDH1 CCA	Src inhibitors demonstrated therapeutic potential in mIDH1 CCA by restoring tumor suppressor functions	[120]

This table summarizes key clinical trials and experimental studies focusing on the role of amino acid metabolism in CCA, highlighting the primary participants, key proteins or mutations involved, experimental content, experimental conclusions, and references.

However, some challenges remain in the above research. The clinical application of nanomaterials is still limited by biosafety issues, and the efficacy of targeted therapies is often affected by secondary resistance. In addition, the complex interplay among genetic mutations, metabolic reprogramming and the immune microenvironment suggests the need for a more comprehensive and personalized therapeutic approach. Future research should focus on elucidating the interplay between metabolic pathways and the immune microenvironment, as well as exploring integrative therapies for CCA. The metabolic reprogramming of amino acids or even the metabolic pathways of the remaining substances could be used to find new therapeutic targets against CCA.

## Data Availability

Not applicable
